# Collaborative development of the Arrowsmith two node search interface designed for laboratory investigators

**DOI:** 10.1186/1747-5333-1-8

**Published:** 2006-07-03

**Authors:** Neil R Smalheiser, Vetle I Torvik, Amanda Bischoff-Grethe, Lauren B Burhans, Michael Gabriel, Ramin Homayouni, Alireza Kashef, Maryann E Martone, Guy A Perkins, Diana L Price, Andrew C Talk, Ruth West

**Affiliations:** 1 Department of Psychiatry and Psychiatric Institute, University of Illinois, MC912, 1601 W. Taylor Street, Chicago, IL 60612, USA; 2 Department of Psychiatry, University of California-San Diego, La Jolla, CA, and Veterans Affairs San Diego Healthcare System, San Diego, CA, USA; 3 Neuroscience Program and Beckman Institute, University of Illinois, Urbana, IL, USA; 4 Professor Emeritus, Department of Psychology and Beckman Institute, University of Illinois, Urbana, IL, USA; 5 Department of Neurology, University of Tennessee, Memphis, TN, USA Current address: Bioinformatics Program, University of Memphis, USA; 6 National Center for Microscopy and Imaging Research and Department of Neurosciences, University of California-San Diego, La Jolla, CA, USA

## Abstract

Arrowsmith is a unique computer-assisted strategy designed to assist investigators in detecting biologically-relevant connections between two disparate sets of articles in Medline. This paper describes how an inter-institutional consortium of neuroscientists used the UIC Arrowsmith web interface  in their daily work and guided the development, refinement and expansion of the system into a suite of tools intended for use by the wider scientific community.

## Background

In the mid 1980s, Don Swanson developed the concept of "undiscovered public knowledge":

"Imagine that the pieces of a puzzle are independently designed and created, and that, when retrieved and assembled, they then reveal a pattern – undesigned, unintended, and never before seen, yet a pattern that commands interest and invites interpretation. So it is, I claim, that independently created pieces of knowledge can harbor an unseen, unknown, and unintended pattern. And so it is that the world of recorded knowledge can yield genuinely new discoveries" [1].

Swanson went on to publish several examples in which two disjoint literatures (i.e., sets of articles having no papers in common, no authors in common, and few cross-citations) nevertheless held complementary pieces of knowledge that, when brought together, made compelling and testable predictions about potential therapies for human disorders [2,3]. Subsequently, his predictions were confirmed by laboratory and clinical studies [4]. One of us (N. S.) began to collaborate with Swanson during the 1990s. Besides publishing more predictions arising from this data-mining approach [5-8], we created a systematic computer-assisted search strategy ("Arrowsmith") and hosted a demonstration website for conducting Arrowsmith searches [9-11].

Initially, Swanson emphasized the so-called one node search, in which one begins with a single literature (e.g., that dealing with a disease) and searches for a second unknown literature having complementary knowledge (e.g. that dealing with potential therapies). In a sense, the one node search is a way of generating new hypotheses [9]. However, it was soon realized that the two node search is more generally applicable and better aligned to the information practices of most biomedical investigators: In this case, user specifies two different literatures A and C which are generally non-overlapping, but are known or hypothesized to have some biologically relevant relationship. The Arrowsmith tool makes a list of words and phrases B that occur in common in the titles of the two literatures, and (after filtering via a "stoplist") presents this so-called B-list to the user. For each B-term, the user can juxtapose the titles containing A and B with those containing B and C, and thereby can more easily judge if the B-term is likely to represent a biologically-meaningful link between A and C. In a sense, the two node search is a way of assessing and prioritizing user-defined hypotheses [9,10].

Arrowsmith is arguably the best established system for carrying out data mining of the biomedical literature, having been widely analyzed, replicated and discussed by the information science community [e.g., [12-24]]. However, as of 2000, it was not clear whether most biomedical bench scientists wanted or needed the kind of information that Arrowsmith could provide. Would they find routine occasions for using such a sophisticated tool? Furthermore, it took many hours to carry out a single search, including crafting Arrowsmith queries, navigating the website, and analyzing the results. Would typical biomedical investigators be sufficiently motivated to learn to carry out Arrowsmith analyses? Would they uncover significant findings that affect their experiments or suggest new research directions? In the present paper, we discuss how field testers interacted with the Arrowsmith software and have collaboratively guided development of the system, including new and (to us) unanticipated directions. A separate paper describes how the Arrowsmith interface and underlying infrastructure have evolved [25]. A third paper (in preparation) will employ field testers' searches as "gold standards" for quantitative modeling of B-terms and implicit information linking two literatures.

## Methods

A five-year Human Brain Project Phase I project was initiated (May 2001) at Univ. Illinois-Chicago to test the feasibility of training biomedical investigators to use the Arrowsmith tool. Arrowsmith was envisioned as comprising a computer-assisted search strategy [9,10], not simply as a stand-alone software product. An important component of the project was to include a number of field testers located within large, multi-disciplinary neuroscience research groups. These sites all had active neuroinformatics research programs in addition to laboratory and/or clinical investigations, and they were chosen to represent a diversity of types of experiments, techniques and data including electrophysiology, biochemistry, electron microscopy and human imaging studies. Each site had a PI who chose one or a few of their group to serve as field testers. These represented a diversity of job descriptions and roles, including graduate students (Lauren Burhans, Alireza Kashef), postdocs (Andrew Talk, Amanda Bischoff-Grethe, Diana Price), staff scientists (Guy Perkins), and principal investigators (Ramin Homayouni, Neil Smalheiser).

The goal of this consortium was to make scientific discoveries, publish papers and identify new directions – typical for a research consortium anywhere, but quite different from a typical software development or evaluation project. We did not recruit human subjects or study their behavior on standardized tasks. Rather, the field testers themselves were the ones choosing freely what searches to perform, and were the ones observing the outcomes. Indeed, despite the use of the third-person in this paper, the field testers are co-authors and have actively contributed to the summarization of the findings. By having field testers conduct Arrowsmith two node searches in the context of their normal work-flow, we hoped to identify the kinds of situations in which opportunities arise for linking different literatures, and to document the various strategies that scientists utilize for handling this type of issue.

**Figure 1:**
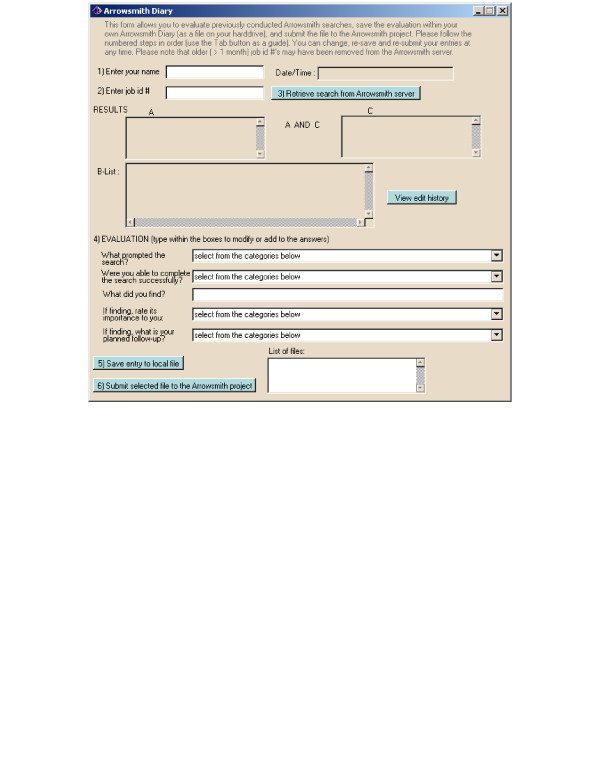
**Electronic notebooks employed by the field testers**. Shown are screenshots of the software used by the field testers to record and submit their evaluations. A templated form for evaluating Arrowsmith searches.

**Figure 2:**
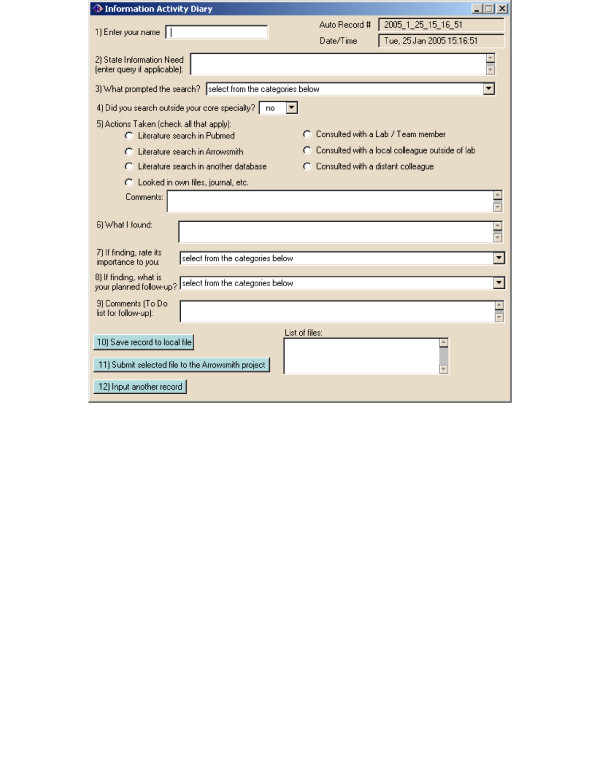
**Electronic notebooks employed by the field testers**. Shown are screenshots of the software used by the field testers to record and submit their evaluations. A templated form for general information seeking activities. The notebook served as an archival system and a follow-up reminder for the field testers, and as a guide for discussion.

During the first year, the team was assembled, the UIC Arrowsmith website was programmed for a multi-user environment, and the semantic category filter was implemented for B-terms (see below) [25]. Searches began in earnest in 2002. Two-day workshops for field testers were held in September 2003 and 2004, as orientation, tutorial and all-hands meetings. Each field tester was given an electronic notebook to record opportunities for conducting Arrowsmith searches (fig. 1), whether they arose from laboratory experiments, from attending seminars, from writing papers, or from discussions with others; and to record the details of completed Arrowsmith searches (fig. 2). Notebook entries were sent to UIC by email or ftp. Weekly phone calls were made by the Project Manager to each field tester to monitor the course of their scientific work, to learn more about completed searches, to suggest alternative ways of searching, to receive suggestions for improving the web interface, and to document follow-up of completed searches. (Although the reason was documented for each Arrowsmith search, and its follow-up, no attempt was made to reconstruct the entire process of scientific activity surrounding a search.) Finally, a number of unsolicited comments and suggestions were received from public users of the Web interface, by email. Over 125 different searches were entered and analyzed.

The Arrowsmith website/user interface and the underlying databases and algorithms have been modified almost continually in response to the needs of the field testers, continuing to the present day. Moreover, the field testers and their projects are highly diverse. Because of this, the present paper focuses on the issue of how field testers contributed to development of the tool, and makes no attempt to assess the performance of the Arrowsmith tool at any given point in time. Despite the fact that the two node searches were topically diverse, we found that B-terms identified as useful for linking in each search reliably tended to share certain features. Thus, although the present observations are qualitative they are not simply subjective – the two node searches included enough objective data to serve as a basis for quantitative modeling (ms. in preparation).

Particularly during the first few years of the study, we were worried about the possibility that field testers might be unduly influenced or biased towards conducting certain kinds of searches or following them up in a particular manner. For this reason, their primary contact for formulating searches was V. T., who has a PhD in Engineering Science but no background in biomedical science. This restriction was relaxed over time as we acquired sufficient data, and as field testers became engaged in collaborative scientific work across sites.

A separate group of social informatics researchers, who are analyzing the broader aspects of scientific information-seeking needs and behavior, has also studied many of the Arrowsmith field testers (among other volunteers) as human subjects, using interviews, site visits and documentary evidence. Their research has been designed, funded and carried out independently of the Arrowsmith Project, and is being published separately [26,27].

## Results

### 1. The initial search interface and logistics of conducting searches

We initially planned to develop a user-friendly interface at UIC that would connect to the software created by Don Swanson for conducting Arrowsmith one node and two node searches [53]. However, Don continually modified his website in the light of his own research, making it difficult to maintain a stable connection to UIC. Internet transfer time was a major problem, as it was necessary to download articles from PubMed [54] to the user's computer and then upload the files to kiwi. Moreover, when running a search via PubMed one could only retrieve 10,000 articles in each literature.

Therefore, a non-mirror website was created at UIC with a completely different user interface and back-end [55], that focused on two node searches exclusively. Because all field testers used PubMed for MEDLINE searching, the PubMed search box was imported into the Arrowsmith site so that it felt to the user like the two searches were being conducted via PubMed itself. No downloading or uploading of files was necessary. Once the A and C queries had been entered and approved by the user, the web server automatically computed a B-list and presented this to the user for further filtering and/or display of AB and BC titles. To further speed queries, a local customized copy of MEDLINE was created in database form so that we only needed to retrieve article IDs from PubMed, not the full records. Only 3 minutes or less were needed to input a search and obtain a "raw" B-list.

All users were able to obtain a B-list successfully, without the need for any significant training. However, the raw B-list – containing hundreds to a few thousand B-terms displayed in alphabetical order – was excessively long for most field testers to scan. One of us (N. S.) had dealt with this head-on by taking separate print-outs of AB and BC titles and juxtaposing them manually over copious cups of café latte while sitting at campus coffeehouses. However, most field testers lacked the sufficient desire for caffeine, so we implemented two filtering strategies that greatly reduced the size of the B-list without greatly reducing the number of interesting links:

First, our colleague Marc Weeber made a list of all terms (words and up to three word phrases) that appear in titles of MEDLINE papers and ran these through the NIH MetaMap program [MMTx version 2.0] to assign each term to one or more semantic categories, if possible, as defined by the Unified Medical Language System (UMLS) [28,29]. He then grouped the 134 semantic categories into ~15 super-categories as outlined in [30]. Users could select terms belonging to any of the super-categories or to any of the individual semantic categories therein; alternatively, they could retain all terms that mapped to at least one semantic category while discarding terms that failed to map at all. This feature was very popular since in most cases the users knew what type of terms they were looking for ahead of time. However, the MetaMap program is not infallible in mapping isolated terms (especially since it is designed to employ information from surrounding text), and certain terms were particularly poorly represented in the UMLS, including neuroanatomical terms and protein and gene names. Therefore, the NeuroNames vocabulary [31] and the Tanabe-Wilbur list of protein and gene names [32] were added as separate look-up lists, that were treated as semantic categories and could be selected by users either individually or as part of semantic super-categories. (During the course of this study, the NeuroNames vocabulary was formally incorporated into the UMLS [33].)

**Figure 3:**
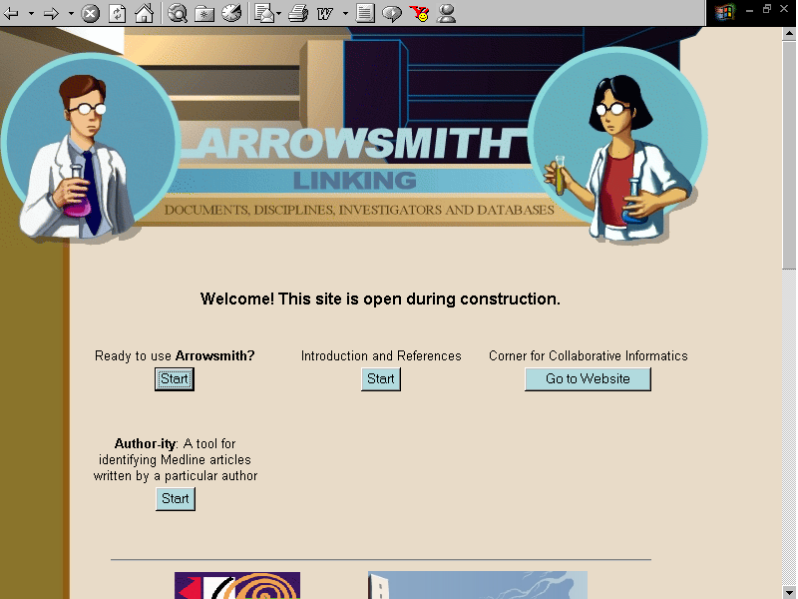
**Screenshots of the Arrowsmith web user interface that was employed by field testers during most of the two node searches described in this paper**. This panel demonstrates a search to construct a list of diseases that are known to be characterized by the presence of both retinal detachment and aortic aneurysm, but not necessarily in the same patient (see Results). The Arrowsmith home page.

**Figure 4:**
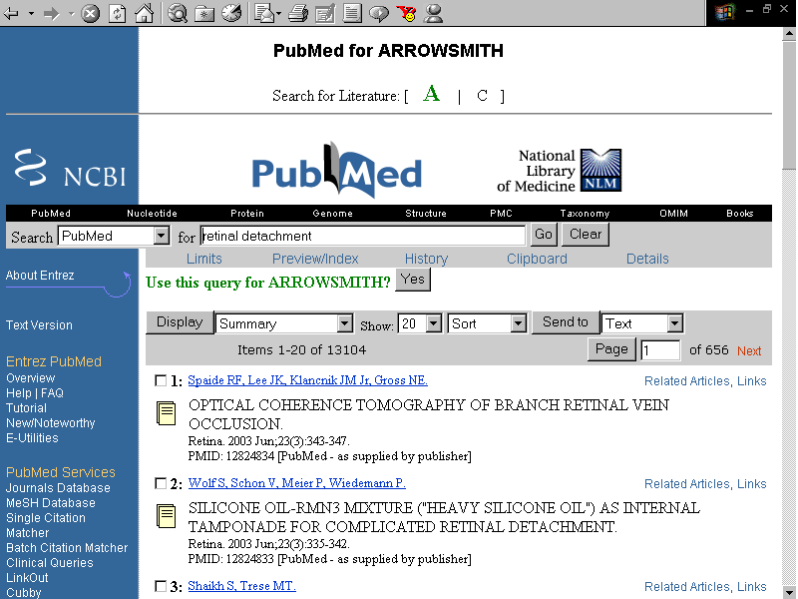
**Screenshots of the Arrowsmith web user interface that was employed by field testers during most of the two node searches described in this paper**. This panel demonstrates a search to construct a list of diseases that are known to be characterized by the presence of both retinal detachment and aortic aneurysm, but not necessarily in the same patient (see Results). Entering the first search, defining literature A: [retinal detachment].

**Figure 5:**
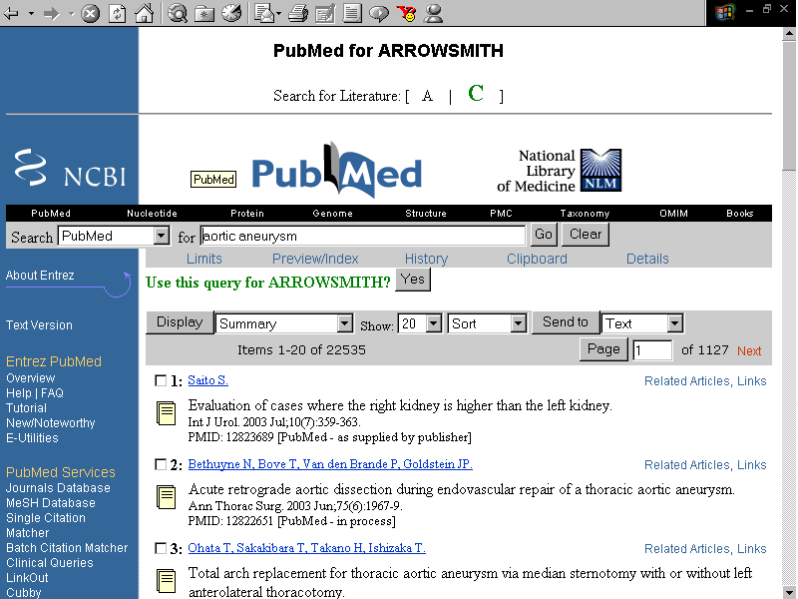
**Screenshots of the Arrowsmith web user interface that was employed by field testers during most of the two node searches described in this paper**. This panel demonstrates a search to construct a list of diseases that are known to be characterized by the presence of both retinal detachment and aortic aneurysm, but not necessarily in the same patient (see Results). Entering the second search, defining literature C: [aortic aneurysm].

**Figure 6:**
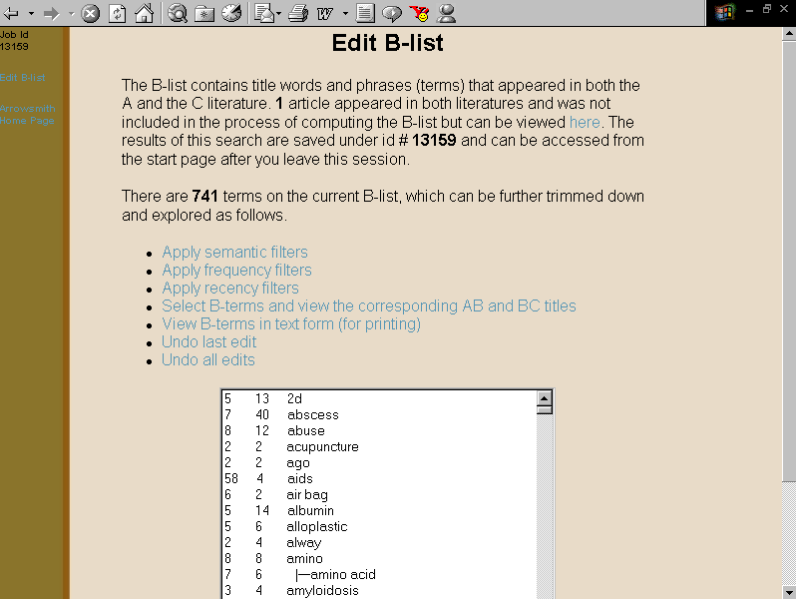
**Screenshots of the Arrowsmith web user interface that was employed by field testers during most of the two node searches described in this paper**. This panel demonstrates a search to construct a list of diseases that are known to be characterized by the presence of both retinal detachment and aortic aneurysm, but not necessarily in the same patient (see Results). The raw B-list, containing 741 terms that are shared between the titles of the A and C literatures. (Note that only one paper in MEDLINE mentioned both retinal detachment and aortic aneurysm in the title.)

**Figure 7:**
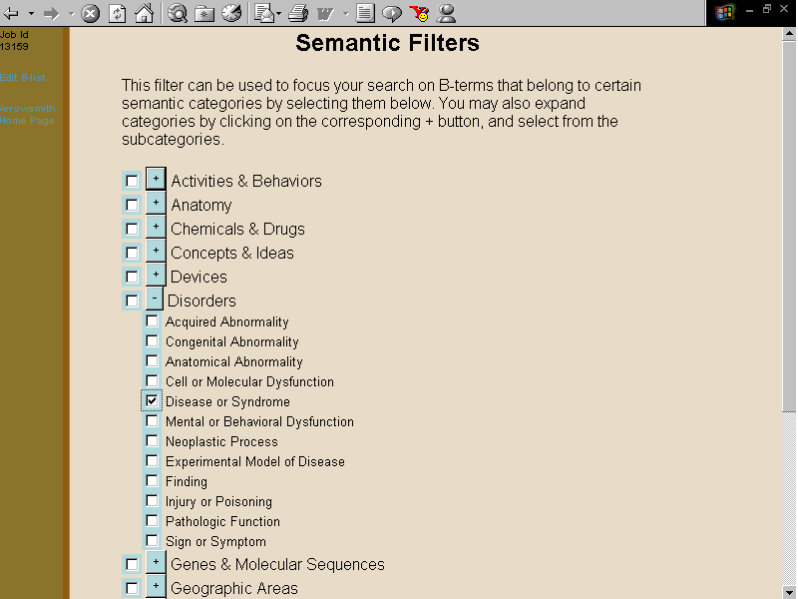
**Screenshots of the Arrowsmith web user interface that was employed by field testers during most of the two node searches described in this paper**. This panel demonstrates a search to construct a list of diseases that are known to be characterized by the presence of both retinal detachment and aortic aneurysm, but not necessarily in the same patient (see Results). The semantic filter, selecting only terms in the category of "disease or syndrome."

**Figure 8:**
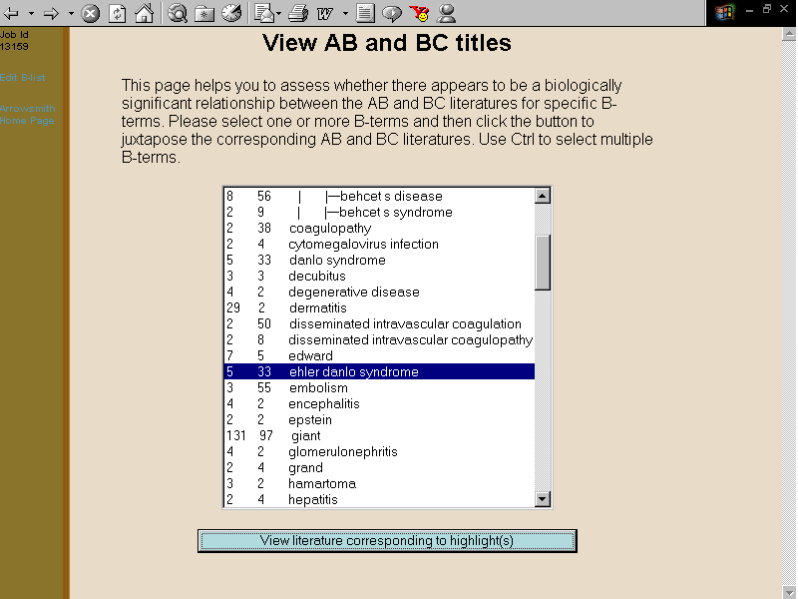
**Screenshots of the Arrowsmith web user interface that was employed by field testers during most of the two node searches described in this paper**. This panel demonstrates a search to construct a list of diseases that are known to be characterized by the presence of both retinal detachment and aortic aneurysm, but not necessarily in the same patient (see Results). The filtered B-list, showing the user selecting a particular B-term, "Ehler Danlo syndrome" for further inspection. (The correct term is Ehlers-Danlos but the final -s was dropped when terms were stemmed during processing of the B-list. In our experience, stemming rarely, if ever, leads to confusion among users.)

**Figure 9:**
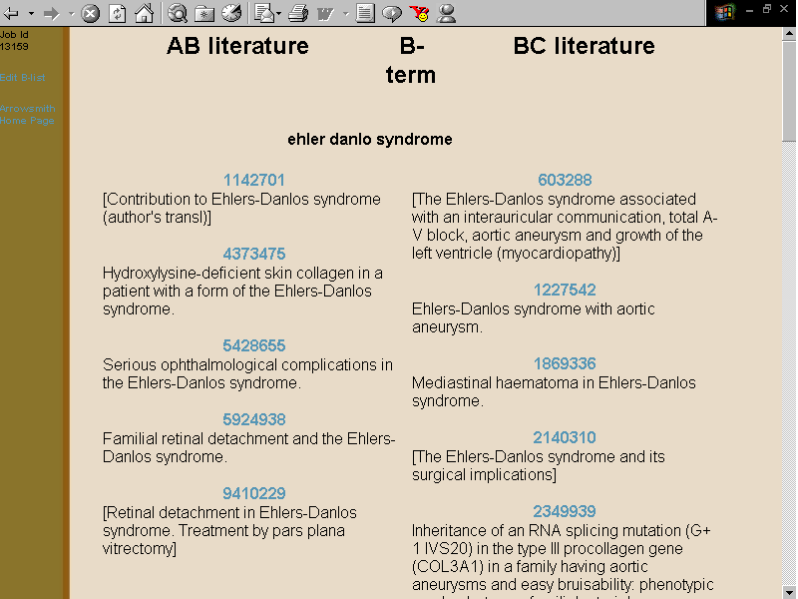
**Screenshots of the Arrowsmith web user interface that was employed by field testers during most of the two node searches described in this paper**. This panel demonstrates a search to construct a list of diseases that are known to be characterized by the presence of both retinal detachment and aortic aneurysm, but not necessarily in the same patient (see Results). The AB and BC titles juxtaposed to each other. That is, titles containing both retinal detachment and Ehlers-Danlos are placed next to titles containing both Ehlers-Danlos and aortic aneurysm. Simple inspection of the titles confirms that Ehlers-Danlos syndrome is, indeed, characterized by both retinal detachment and aortic aneurysm. One can easily inspect other B-terms in the same manner.

Second, a frequency filter was implemented which, as a default setting, removed all B-terms that appeared in only one paper in either literature [for literatures over 1000 articles in size]. This removed about ¾ of B-terms, yet users judged informally that very few "interesting" terms were lost. (This has subsequently been evaluated more rigorously [ms. in preparation].) Most of the field tester searches were conducted after the semantic and frequency filters were implemented (fig. 3, 4, 5, 6, 7, 8, 9). We also embarked on a long-term research agenda to identify additional filtering and ranking procedures; a total of 8 filters have been implemented to date [25]. Initially a short list was displayed of the top ~50 predicted relevant B-terms, but presently the Arrowsmith website displays all B-terms ranked in order of the likelihood that they will provide relevant linking information across the two literatures.

### 2. Information-seeking needs and strategies of field testers

We originally expected that two node searching would be pursued in a strategy consisting of two distinct steps [9-11]. First, it was expected that field testers would define an hypothesis that involves finding possible links between two disparate fields of inquiry A and C, which show little or no overlap (in terms of having few or no papers in common). We had recommended that users should first examine a broadly defined PubMed search of "A AND C" to find and analyze any direct literature that might be present (including use of related MeSH terms, and instances of A and C and synonyms thereof in both title and abstract fields). Second, after understanding what is already known about A and C in context, they would then carry out an Arrowsmith two node search using narrowly defined queries on A and C (for example, restricting the search to instances of A and C in the title fields). Nevertheless, we did not constrain the type or manner of searches performed by field testers, but encouraged them to "play" with possible uses in the course of their scientific work.

To our knowledge, none of the field testers ever followed the recommended scenario. In fact, regardless of their seniority or training, none of them carried out basic PubMed searches in the manner recommended by seasoned information scientists either [34]. Rather, they quickly scanned the first one or two pages of articles returned with the goal of obtaining a few, recent, relevant papers: a) They were not concerned with maximizing either precision or comprehensiveness of the search; b) they did not spend a lot of time crafting the initial query carefully, nor did they tend to modify and re-enter queries; c) they generally did not use advanced options such as the Details button to see how the search terms were processed. Little attention was given to ambiguous terms (e.g., "cold" can refer to cold temperature, the common cold or chronic obstructive lung disease), or to terms strung together with multiple AND, OR or NOT operations (which raises the possibility of retrieving too many or too few papers). (See discussion in Appendix 1.) Once we recognized that the behavior of field testers in conducting two node searches was consistent with their approach to simple PubMed searches, field testers were instructed NOT to attempt to find all possible complementary information inherent in a given Arrowsmith search, but instead to carry out relatively quick, short, focused searches that answered specific questions arising in the course of their research.

By asking field testers to employ the two node search as part of their normal work flow (Table 1), numerous applications appeared that were previously unanticipated (see below). Assessing and prioritizing hypotheses was primarily carried out by principal investigators or those engaged in designing experiments and writing grant proposals. Graduate students (and to a lesser extent, postdocs) expressed the feeling that their job description was limited to carrying out experiments within the framework set by their supervisors. Thus, they did not feel comfortable looking for links that might send them off exploring new disciplines or using new techniques. Even if links of interest were found during the course of an Arrowsmith search, they felt they had limited influence on the design of future experiments carried out in their laboratory groups. For this cohort, Arrowsmith searching was largely restricted to the more concrete tasks of information seeking behavior, for example, obtaining information that might contribute to the discussion section of papers being written, or assessing whether an unexpected, anomalous finding in the laboratory warranted follow-up (Table 1).

**Table 1:** Common information-seeking tasks of field testers.

• Learn a field for the first time • keep up with advances in one's own field • find specific items of information (facts) • browse, keep up with science in general • find information in a different field relevant to one's own, or related to a collaboration • prepare a paper, grant proposal or review article • follow-up on an unexpected finding • assess an hypothesis or new idea

### 3. "Classic" Arrowsmith two node searches designed to assess and prioritize hypotheses

On numerous occasions two node searches did, indeed, identify promising new research directions that led to the planning or execution of new experiments, or that led to new submitted and even funded grant proposals. Five examples will be summarized below to emphasize their range and diversity (see Appendix 2 for 6 additional examples).

#### Example 1

R. H. found an unexpected, previously unreported interaction between two proteins in a biochemical affinity screen: ubiquitous mitochondrial creatine kinase (uMtCK) and the cytoplasmic tail of amyloid precursor protein (APP) [35]. In order to learn whether this interaction might have important physiological consequences, particularly in Alzheimer disease (a condition with disordered APP metabolism), several Arrowsmith searches were performed. A search on [creatine kinase] vs. [Alzheimer] was not helpful, since it pointed to the cytosolic isoform of the enzyme (brain creatine kinase or CKB). A search on [creatine kinase] vs. [amyloid precursor protein] identified several interesting linking concepts including "ischemic." Notably, APP is induced after ischemic insults and transgenic expression of APP in mice increases the vulnerability to ischemic damage. A number of papers indicated that uMtCK plays a critical role in mitochondrial function not only because of its ability for energy transfer from mitochondria but also as a structural component in controlling mitochondrial integrity through its association with mitochondrial proteins porin and adenine nucleotide translocase (ANT). The coupling of uMtCK with ANT is preserved in ischemic preconditioning, a process known to protect against ischemic damage. These results suggested to him the hypothesis that the cytoplasmic tail of APP might be involved somehow in the biosynthesis, mitochondrial targeting or function of mitochondrial creatine kinase. In the laboratory, R. H. found experimental evidence that APP stabilizes the mitochondrial creatine kinase pre-protein both in the cytosol and in the mitochondrion [35]. Based on these preliminary data, a grant application was submitted entitled "Role of APP as a chaperone for mitochondrial proteins."

#### Example 2

D. P. was working with a transgenic mouse that overexpresses human alpha-synuclein as an experimental animal model of Parkinson Disease (PD). Alpha-synuclein is a major component of the pathological hallmark of PD, Lewy bodies. This mouse model mimics certain aspects of PD at both behavioral and neuroanatomical/neuropathological levels, including the formation of Lewy bodies in certain neurons within the brain. The pattern of affected neurons does not correspond to any obvious rationale. However, since metabotropic glutamate receptors, subtype 5 (mGluR5) antagonists are being considered as an adjunctive therapy in Parkinson disease patients, D. P. wondered if alterations in mGluR5 receptor expression might somehow facilitate the formation of Lewy bodies. To see if the literature supported such a causal connection, she conducted a two node search on [metabotropic glutamate receptor OR mGluR] vs. [Lewy bodies]. A number of meaningful potential links were found: For example, mGluR5 stimulation results in tyrosine phosphorylation of ERK1/2 leading to its activation in both neurons and glial cells, and on the other hand, ERK-2 appears to be present in Lewy bodies (LBs) in the brain stem of PD. Another link was via calcium: mGluR5 stimulation increases intracellular Ca^2+ ^levels within neurons, and Ca^2+ ^binding to alpha-synuclein regulates ligand binding and oligomerization, which potentially could modulate formation of Lewy bodies.

The observed links were sufficient to encourage D. P. to carry out experiments mapping the expression of mGluR5 receptors in alpha-synuclein transgenic mice. Prior to conducting the immunolabeling experiments, she conducted behavioral experiments to assess cognitive and motor function(s) which revealed both cognitive and motor deficits in the transgenic animals. The immunolabeling experiments revealed increased mGluR5 levels in the brains of transgenic animals in comparison with non-transgenic control animals. This observation was confirmed using Western Blot analysis. The transgenic brain regions with increased mGluR5 receptors correspond to areas associated with rodent cognitive and motor function (e.g. hippocampus and basal ganglia). She is preparing a manuscript describing these findings and has submitted a K-award application to NIH following up on this work.

#### Example 3

A. T. was studying the role of the hippocampus in discriminative avoidance learning in rabbits. He started from the known fact that theta rhythm is important in hippocampal function and appears to play a role in learning and memory, and he noticed that neurogenesis in the hippocampus has recently been proposed to be involved in memory formation, as well. To see if there were any possible links between these apparently disparate concepts, he performed an Arrowsmith search on [theta rhythm] vs. [neurogenesis]. No articles mentioned both theta rhythm and neurogenesis. One of the B-terms of interest to him was paradoxical (REM) sleep, since this state of consciousness is associated with high theta rhythm activity. The indirect links in the literature suggested that REM sleep stimulates theta rhythm, which activates NMDA receptors, which in turn are critical for formation of new memories. The rate of hippocampal neurogenesis has been found to correlate positively with learning ability in both normal and irradiated animals. (There was a potential discrepancy in the last link of the chain, however: Hippocampal neurogenesis was stimulated by NMDA receptor antagonists given experimentally. This might not be a true discrepancy, since NMDA receptor antagonists are known to produce rebound excitation after they are withdrawn, due to supersensitivity of the NMDA receptors.)

Taken together, the indirect links favored the hypothesis that depriving a rat of REM sleep should decrease the rate of neurogenesis in the hippocampus. At the time, no studies on this hypothesis had been published in MEDLINE. A. T. submitted an animal protocol to test this hypothesis, though resources were not immediately available to support experimental studies. Subsequently, a number of papers have appeared indicating that sleep deprivation (including, but not specific to the REM stage of sleep) decreases cell proliferation in the dentate gyrus and hippocampus, and also suppresses learning-induced hippocampal neurogenesis [36-39].

#### Example 4

During the above study, A. T. also noted that neurogenesis has been documented as occurring not only in the hippocampus, but also in the amygdala (and other areas of the brain) of adult mammals. Whereas the hippocampus is involved in certain types of learning, particularly related to spatial locations of items, the amygdala participates in behaviors with strong emotional content, such as fear conditioning. Insofar as hippocampal neurogenesis has been closely linked to hippocampal-dependent learning, and is **suppressed **during fear conditioning [40], he formulated the hypothesis that neurogenesis in the amygdala should be **stimulated **by fear conditioning. This hypothesis remains to be tested.

#### Example 5

Finally, a striking testimonial came from a web-based public user of the Arrowsmith website, Dr. John Goudreau, a neurologist at Michigan State University, who learned about Arrowsmith at a Society for Neuroscience short course. Dr. Goudreau, an expert on animal models of Parkinson disease, received a flyer from Pfizer inviting physicians to propose research on disorders that might be ameliorated by sildenafil (Viagra). A PubMed search on [sildenafil AND Parkinson disease] revealed no prior studies on the subject. However, an Arrowsmith two node search immediately revealed that sildenafil increases cyclic GMP levels in many cell types, and cyclic GMP (and agents that raise cyclic GMP) are neuroprotective in several model systems. This strongly suggested that there was, indeed, a rationale for examining the effects of sildenafil in animal models of Parkinson disease. Dr. Goudreau wrote a grant proposal to Pfizer that was subsequently funded and is currently ongoing.

### 4. Variant and hybrid searches

Sometimes two node searches were conducted to find information that could have been obtained from a direct PubMed search. For example, users occasionally entered separate A and C queries in order to examine the set of papers present in both A and C literatures (which are listed in a separate window within the web interface). This could have been retrieved via PubMed more simply by entering the query [A AND C]. Nevertheless, using the two node interface provided the option of allowing them immediately to examine the B-terms linking the two literatures, should they wish.

One major use for the two node search was to construct a list of items that are common to two (overlapping or non-overlapping) sets of articles [41]. For example, one of our colleagues wanted to construct a list of diseases that are characterized by both aortic aneurysm and retinal detachment (not necessarily in the same person, and not necessarily described in the same paper). A two node search on [aortic aneurysm] vs. [retinal detachment], with B-terms filtered for the semantic category of diseases, readily gave the desired result (fig. 3, 4, 5, 6, 7, 8, 9).

Another major use of the two node search was to browse articles in a discipline removed from one's own, or to view one literature in light of another context (e.g., a specific disease). In most cases, a field tester was an expert in one field (represented by literature A) and entirely unfamiliar with the second literature (C). Here the goal was to identify the articles in literature C which are most likely to be relevant or helpful to field A. For example, G. P. wanted to browse the literature on mitochondrial complex I – not the entire literature, which contains over 10,000 articles, but only those sharing certain B-terms with the literature on Parkinson's disease.

### 5. One node search opportunities

The one node search is used to find an unknown literature that may contribute to a specific problem area. We encountered a number of situations in which field testers formulated problems that called naturally for one node searches. For example, R.H. devised his own method of relating direct and indirect gene co-occurrence links in the literature [42] to predict a set of proteins that are likely to interact functionally with the reelin signaling pathway, even though they do not co-occur in any paper mentioning reelin. In another case, A. T. was interested in identifying a list of genes that are known to be expressed in the amygdala, that have NOT previously been studied in the context of fear conditioning, yet that could plausibly be expected to play a role in that process.

### 6. Field tester suggestions

Field testers made ongoing suggestions regarding the interface and proposed ways that the interface could be expanded for specialized purposes. Field testers met with V. T. as well as R. W., a graphic designer, resulting in improvements to the web interface: For example, in 2003 a clipboard was added to save selected articles, a flowchart was added to the top of the search pages to serve as a roadmap to the overall search procedure, and a Venn diagram was added to visualize the relative size and overlap of the two literatures. Several field testers expressed the desire to have Arrowsmith encompass bibliographic databases beyond PubMed, so that education, psychology, engineering and computer science papers could be searched too – unfortunately, this was not feasible due to licensing restrictions by the latter services. Finally, several field testers and public web users suggested adding gene-centric extensions of Arrowsmith, such as looking for common features among an entire group of genes identified during a microarray experiment.

## Discussion

The two node search is based on a clear, logical model of hypothesis formation and testing, and follows a systematic multi-step procedure [9,10] – yet we have found surprises at every turn when biomedical investigators were allowed a free hand at employing this tool during the course of their everyday scientific work. The needs of the field testers blurred distinctions among simple information retrieval, hypothesis formation, summarization of a literature, and browsing within an unfamiliar field. In turn, this has led us to re-engineer the web interface and its underlying databases and algorithms, and indeed to re-conceptualize the two node search process.

The first lesson we learned is that the list of B-terms needs to be displayed and assessed within a matter of a few minutes, rather than hours or days. We created a series of 8 filters to allow the searcher to restrict manually the number of B-terms that need to be examined. However, this required a great deal of mental facility and judgment, since each search must be customized. Having a corpus of field tester queries, and the B-terms that were marked as relevant (i.e. indicating meaningful links between literatures A and C), have permitted the development of a quantitative model that permits us to display all B-terms ranked in order of the probability that they are likely to be relevant to some user (ms. in preparation). This now permits the user to scan very quickly down the ranked B-list, though further manual filtering is still available as an option.

The second lesson was that field testers viewed the two node search not as a detailed two-step strategy of exploring all possible relationships between two literatures, but rather as a straightforward extension of simple PubMed searches. In turn, they viewed PubMed searches not as a way to gain comprehensive knowledge of a particular field, but as a means to identify one or a few recent, relevant papers to satisfy a very specific information need [which generally is not fully captured by the query terms entered into the search interface]. Thus, just as the formal Japanese ***chadō ***tea ceremony has given way to dunking a tea bag into hot water, so the Arrowsmith two node search has evolved from beauty to practicality.

Third, it was gratifying to verify that field testers were able on many occasions to employ two node searches to assess and prioritize their hypotheses. Two node searches did affect their work-flow insofar as they suggested new ideas for articles or grant proposals, and suggested or supported new lines of investigation in the laboratory. However, field testers were not interested only in identifying novel unreported links, but sought to identify meaningful links even if they were well known in the literature already. We initially expected that users would primarily be interested in links of a causal nature ("A affects B which affects C"). However, many searches took the form: "find items (concepts, methods, organisms, proteins, anatomical regions, etc.) that have been studied in both literatures A and C". As well, many searches took the form of browsing: "find me the papers in literature C that are most similar to, or likely to be relevant to, the papers in literature A".

These applications greatly increase the potential scope and audience of Arrowsmith, and suggest several avenues for extending the tool. For example, for browsing, one might wish to bypass the B-list display and present users directly with selected article titles that are organized in some easy-to-scan manner. Also, this suggests future extensions for the UIC Arrowsmith website, such as allowing users to conduct two node searches employing B-terms taken from any specified Medline fields (authors in common, affiliations, MeSH, etc.), as well as to carry out two node searches between two sets of full-text papers in PubMed Central. Although the signal-to-noise ratio may be unfavorable to find causal links embedded in text efficiently, many items are only encoded in full-text: For example, a two node search on full-text articles could readily reveal references cited, reagents employed or persons acknowledged in both literatures.

Finally, our experience with field testers has identified a number of additional information-seeking needs for which we are constructing new tools that are freely available [55]. The Author-ity project [43] is disambiguating author names in Medline, to permit users to cluster articles according to the specific individuals that authored them, and to permit a better understanding of scientific publishing behavior and collaboration networks. The Anne O'Tate tool allows users to summarize features of a PubMed search outcome very quickly (e.g. authors publishing in that set of articles listed by frequency, journals, terms used in title or abstract, Medical Subject headings, etc.), and permits them to drill-down further without needing to revise and resubmit their queries. The WETLAB project is creating a very simple, searchable, open source, cross-platform electronic laboratory notebook. We believe that text-mining has the potential to make a significant contribution to the processes of scientific discovery and collaboration.

## Competing interests

The author(s) declare that they have no competing interests.

## Authors' contributions

N. S. conceived and directed the study, interacted regularly with field testers, and conducted orientation sessions and tutorials. V. T. programmed the electronic notebook, interacted regularly with field testers, and kept documentation on searches and their follow-up. He also programmed, maintained and upgraded the web interface and the underlying software and databases. A. G., L. B., R. H., A. K., G. P., D. P., and A. T. used the two node search tools during the course of their scientific work and provided feedback, critiques and suggestions. R. W. interviewed field testers and analyzed the workflow and design requirements of the web interface. M. G. and M. M. supervised field testers in their scientific studies and double-checked the adequacy and coverage of the manuscript.

## Appendix 1. Search strategies of field testers for everyday information needs and tasks

The field testers were given both hands'-on and tutorial sessions on conducting basic PubMed searches and Arrowsmith two node searches, and Don Swanson wrote a tutorial for them discussing strategies for MEDLINE searching [34]. Nevertheless, regardless of their knowledge, training, or experience – and regardless of whether they were graduate students or seasoned principal investigators – field testers rejected the recommended strategy of carefully crafting queries and successively modifying them in an attempt to formulate the optimal query for a given search. Rather, an initial query was posed and then the first one or two pages of retrieved results were scanned for one or a few relevant articles.

From the viewpoint of an information scientist, such a strategy might appear to be ignorant, sloppy or even reckless – akin to driving without seat belts or even a steering wheel! And yet, the field testers were familiar with the proper handling of complicated, sophisticated tools such as electron microscopes, electrophysiological rigs, microarrays and fMRI imaging machines. Thus, their search behavior must be acknowledged as a deliberate strategy, one which may be judged as more or less effective according to how well it answers the needs of users, but which cannot be disregarded out of hand.

Further research is needed in order to understand the search behavior of field testers: One possibility is that because PubMed and most current information retrieval systems are designed to retrieve comprehensively ALL relevant papers on a given topic, they are not well aligned with the more discrete needs of the typical user. A second possibility is that the ease of "Googling" on the web has created an expectation that searching should result in instant gratification. Because searchers had intimate knowledge of their own fields and were familiar with appropriate and specific search terms to use in their queries, perhaps this allowed them to craft their initial query in such a manner as to get acceptable results in most cases. A third possibility is that users intuitively realize that it is difficult, if not impractical, to craft a query such that it captures the full context of a given search. Finally, it may be that current information retrieval systems give relatively poor performance even when given an "optimal" query [44]. In any of these cases, quickly scanning the search output (keeping the context in mind) may be the most efficient course of action.

To our knowledge, no studies have systematically examined the PubMed search behavior of students or professional scientists under free-range conditions – that is, when the task is chosen freely by the user, and when the outcome is evaluated in terms of whether the information retrieved was adequate for the question asked. Such studies might uncover user optimization principles that go beyond precision and recall, or at least may give clues to ways that search interfaces can become better aligned with the needs of their users.

## Appendix 2. "Classic" Arrowsmith two node searches designed to assess and prioritize hypotheses

Five examples are given in the body of the paper, and six additional examples are discussed here.

### Example 6

A number of searches were directly motivated by trying to understand unanticipated findings made by the field testers in the course of their ongoing studies. For example, previous experiments had demonstrated that plasticity of neural activity within the medial geniculate nucleus, during discriminative avoidance in rabbits, was much greater in the medial division of this nucleus as compared to the ventral division [45]. To identify factors that may account for the difference in neuronal responses (especially, proteins expressed differentially in these two regions), A. K. undertook a number of two node searches, including a search on [(medial geniculate OR medial geniculate bodies) AND ventral] vs. [(medial geniculate OR medial geniculate bodies) AND "medial division"] looking for terms in the category of gene or protein names that might be related to neural plasticity or learning. He found that a particular subtype of NMDA receptor (NR2A) is more abundantly expressed in the medial division, whereas NR2B is expressed mainly in the ventral division [46]. This was interesting since, in other brain regions, NR2A had been implicated in LTP (a form of plasticity related to that seen in the medial geniculate) whereas NR2B was implicated in LTD. Thus, this suggested the hypothesis that NR2A mediates neuronal activation during discriminative avoidance as well.

### Example 7

D. P. was attempting to assess the potential relevance of astrocytes in the Parkinson's disease process, and in the course of conducting a number of two node searches, found two references reporting that (1) exposure of astrocytes to thrombin reduces levels of the metabotropic glutamate receptor, and (2) mGluR5 metabotropic glutamate receptor activation enhances the activities of two types of Ca^2+^-activated K^+ ^channels in rat hippocampal astrocytes. Because astrocytic Ca^2+^-activated K^+ ^channels have been suggested to participate in the normal coupling of neuronal activity to blood flow, disruption of this process might have implications for astrocytic-neuronal homeostasis. The successful application of mGluR5 antagonists in a subgroup of PD patients further suggests that further study of the link between mGluRs and glia are warranted. The first reference is also interesting because of reports that thrombin signaling in response to CNS injury induces thrombospondin release, and that thrombospondin released by astrocytes induces synaptogenesis. Taken together, D. P. hypothesized that astrocytic thrombospondin/thrombin signaling may be an early response to, or modulator of, PD pathogenic signals.

### Example 8

After a paper by Sheng's group appeared reporting that mitochondrial activity is rate-limiting for formation of new dendritic spines and synapses following neuronal stimulation [47], G. P. and N. S. jointly brainstormed to identify promising "gaps" in current knowledge regarding potential roles for mitochondria in neuronal plasticity and learning. Local regulation of protein synthesis at dendritic spines for synaptic plasticity is under intensive investigation by many laboratories. Yet, we noticed that local regulation of mitochondrial protein synthesis was almost totally ignored in this context (one study had ruled out mitochondrial protein synthesis as a factor in regulating early-onset long-term potentiation (LTP)). Yet a two node search on specific inhibitors of mitochondrial protein synthesis [chloramphenicol OR tetracycline] vs. [learning OR memory] revealed that these drugs have been shown to inhibit specific phases of memory in vivo [e.g., [48]]. This suggests that mitochondrial protein synthesis might be involved in regulating LTP, dendritic spines or synaptic plasticity, particularly in late or sustained phases of consolidation and growth; it may be worthwhile to test experimentally whether chloramphenicol or tetracycline affect these processes.

### Example 9

After reading a newspaper article about treating restless legs syndrome with dopaminergic medications, A. G. sought to catalog the similarities between restless legs syndrome (RLS) and bruxism (teeth grinding). Although there is direct literature discussing both conditions, a two node search was also judged to be helpful in revealing similarities: Both restless legs syndrome and bruxism can be aggravated by stress. About 10% of RLS sufferers also have bruxism. In rat, dopamine agonists increase bruxism. And, both are comorbid factors in Tourette syndrome (a basal ganglia disorder) as well as in Parkinson disease patients.

### Example 10

N. S. routinely employed two node searches during both the experimental and writing phases of his research. For example, while testing the hypothesis that calpain would cleave and activate dicer near synapses [49], he carried out a two node search on [calpain] vs [postsynaptic density] to construct a list of other proteins known to be cleaved by calpain and located at the postsynaptic density. Another study, which demonstrated that genomic repeats contributed to certain microRNA genes and their targets [50], motivated a search on [Alu] vs. [microRNAs], which in turn led to a computational study predicting that Alu repeats within 3'-UTRs of messenger RNAs serve as target regions for mammalian microRNAs [51]. A NIH grant proposal was submitted to test this hypothesis experimentally.

### Example 11

Extracellular double-stranded RNA is a signal for triggering systemic RNA silencing in C. elegans; several of the proteins needed for transport of double-stranded RNA are conserved in mammals [52] and expressed in mammalian brain. Might double-stranded RNA have a role in physiologic signaling among neurons? N. S. wondered if perhaps extracellular double-stranded RNA applied to brain tissue might cause cells to secrete cytokines that, in turn, might stimulate neurite outgrowth. A two node search on [dsRNA] vs. [neurite outgrowth] was carried out and filtered for terms in the category of Immunologic Factor. A handful of cytokines were identified in the B-list (interferon gamma, interleukin-1, interleukin-6 and tumor necrosis factor). Interferon gamma appeared promising as a putative factor, insofar as dsRNA applied to cells can elicit interferon gamma, and since other papers reported that (in a different context) inferferon gamma can stimulate neurite outgrowth.
